# Me, we, they: identifying the key stressors affecting the dental team

**DOI:** 10.1038/s41415-025-8645-z

**Published:** 2025-08-08

**Authors:** Ian Mills, Jennifer Knights, Fiona Ellwood, Robert Witton, Linda Young, UK Dental Team Mental Health Research and Implementation Group

**Affiliations:** 736513014155233694589 University of Plymouth, Peninsula Dental School, Plymouth, United Kingdom; 081429578268608379806 NHS Education for Scotland, Edinburgh, UK; University of Dundee, Dundee, United Kingdom; 379134042197391891894 Chair, Mental Health Wellness in Dentistry Group, United Kingdom; 997940520353436025716 NHS Education for Scotland, Edinburgh, United Kingdom

## Abstract

**Introduction** The mental health and wellbeing of the dental workforce is essential in providing oral healthcare services which are sustainable, safe and of the highest quality. Yet, there remains a lack of qualitative studies exploring the factors that negatively affect wellbeing in dentistry in the United Kingdom, not least in regard to the views and experiences of the wider dental team.

**Aim** The aim of this paper is to identify and explore the factors that contribute to stress and burnout within dental teams as reported through the MINDSET U.K. Survey 2023.

**Method** Qualitative data were collected in an online questionnaire which provided an opportunity for respondents to provide a free-text response. Following an inductive approach, thematic analysis was used to synthesise the findings.

**Results** In total, 1,507 responses were received, of which 287 included a valid free-text response. The sample included 203 dentists, 69 dental care professionals, 13 practice managers/receptionists and two respondents who did not select a professional group. Six themes were identified from the data: workload; NHS system; regulatory compliance, patient complaints and litigation; financial pressures; leadership and management; and self-worth.

**Conclusion** Current reactive approaches to dealing with the mental health and wellbeing of dental healthcare workers are insufficient. Measures need to be urgently developed and implemented to reduce or mitigate the contributing factors at the macro (system) level. These need to be considered as a priority in order to create the working conditions necessary to allow all members of the dental team to develop, flourish and feel valued.

## Key points

- A significant proportion of the dental workforce are unhappy and unfulfilled in their role, with concerning levels of despair among some respondents who appear to be suffering serious mental health issues, including suicidal ideation.
- The main stressors identified can be largely attributed to macro-level factors, suggesting that the contributors to poor mental health and wellbeing predominantly sit outside of the locus of control of most individuals.
- Current reactive approaches to dealing with the mental health and wellbeing of dental healthcare workers are insufficient to address the macro-level factors.
- Prioritisation of policy and system-wide interventions, in addition to individual and team-based solutions, are urgently required.

## Introduction

It is widely acknowledged that dentistry is a stressful occupation,^1^^,^^2^^,^^3^^,^^4^ with a recent report suggesting that ‘the high levels of self-reported stress, burnout and psychological distress are a serious concern to the profession'.^1^ All members of the dental team, irrespective of role or setting, are affected and this can have a significant impact on the individual, the dental team and on patient care.

Stress, as defined by the World Health Organization (WHO), is ‘a state of worry or mental tension caused by a difficult situation'.^5^ Stress is a natural human response when challenged and can have a positive impact leading to improved performance. However, long-term stress can lead to fatigue, emotional exhaustion, poor mental health and burnout.^6^ Burnout is defined by the WHO as an occupational phenomenon due to ‘chronic workplace stress which has not been successfully managed'.^7^ It is characterised by three elements: exhaustion, remoteness or negativity towards the job, and reduced professional efficacy.^7^

The mental health and wellbeing of the dental workforce is essential in providing oral healthcare services which are sustainable, safe and of the highest quality. Anxiety, stress, or mental health-related issues can impact significantly on clinical performance, and although the evidence in dentistry is sparse, research in other areas of healthcare demonstrates a strong link between stress and impaired surgical competence and communication,^8^ with issues of burnout resulting in compromised work performance, absenteeism^9^ and worsening patient safety.^10^^,^^11^ Reports on stress within dentistry have identified numerous systemic stressors, including: time limitations; working in the NHS (National Health Service); working environment and conditions; fear of regulation and litigation; unrealistically high workload; and patient issues.^2^^,^^3^^,^^12^^,^^13^^,^^14^^,^^15^ The risks of litigation or regulatory chastisement have increasingly been recognised as major stressors for many, with dentists reported to be operating ‘under constant fear of persecution'.^1^

The majority of research previously undertaken on stress in dentistry pertains to dentists and has failed to include the views and experiences of the wider dental team. This paper draws upon qualitative data collected through the Mental health IN Dental SETtings U.K. Project (MINDSET U.K) Survey 2023, designed to evaluate current levels of burnout, depressed mood, experienced trauma, and preparedness to provide quality care in dental teams in the United Kingdom (UK).

## Aim

This project was initiated by the UK dental team mental health research and implementation group, comprising experts in mental health and dentistry. The aim of the research presented within this paper was to identify and explore the factors that contribute to stress and burnout within the dental team.

## Methods

### Study design

The findings reported within this paper are part of a wider study (MINDSET U.K.) which involved a UK-wide, cross-sectional survey of all members of the dental team. The anonymous online survey collected quantitative and qualitative data on current levels of burnout, depressed mood, experienced trauma, and preparedness to provide quality care in dental teams in the UK. The questionnaire was hosted on Microsoft Forms by NHS Education for Scotland (NES) and was open from 19 April 2023 to 30 June 2023.

Members of the dental team across a wide range of dental settings in the four countries of the UK were invited to complete the questionnaire. Settings included the General Dental Service (GDS), both independent and corporate, Public/Community Dental Service (PDS/CDS), Hospital Dental Service (HDS), armed forces, prison service, public health and higher education institutions. The questionnaire was disseminated widely according to country-specific strategies overseen by the chief investigator for each country. This included dissemination via the NHS, national organisations, specialist associations and societies, professional networks, and social media.

### Data analysis

Qualitative data were gathered using a free-text box and analysed using an inductive thematic approach to identify themes within the data.^16^ Initial codes were generated to systematically identify interesting and salient features of the data in relation to the specific research question. These codes were collated and developed into potential themes. A group formed of four of the project investigators (JK, FE, IM, RW) met to discuss the thematic development and agree themes and subthemes. This process acted to ensure trustworthiness in terms of credibility.^17^^,^^18^

### Ethics, governance and data protection

The study was classified as an evaluation of service impact on service delivery staff and therefore did not require institutional review or NHS Research and Development review and approval. This outcome was confirmed by the following bodies: NES, NHS Tayside, Health Education and Improvement Wales, King's College London and the Office of Research Ethics Northern Ireland.

A participant information sheet (PIS) containing detailed information about the study was provided. Respondents were asked at the outset of the questionnaire to confirm they had read and understood the PIS and understood that the data collected was anonymous. A positive response to both questions and completion of the questionnaire implied consent. The PIS signposted respondents to their doctor or NHS Practitioner Health should they wish to speak with someone about their mental health. A weblink to various charitable helplines was also provided.

## Findings

Completed survey responses were received from 1,507 respondents, which included 287 valid free-text responses. The qualitative sample included a wide range of team members: 203 dentists, 69 dental care professionals (DCPs), 13 practice managers/receptionists and two undeclared.

**Fig. 1:**
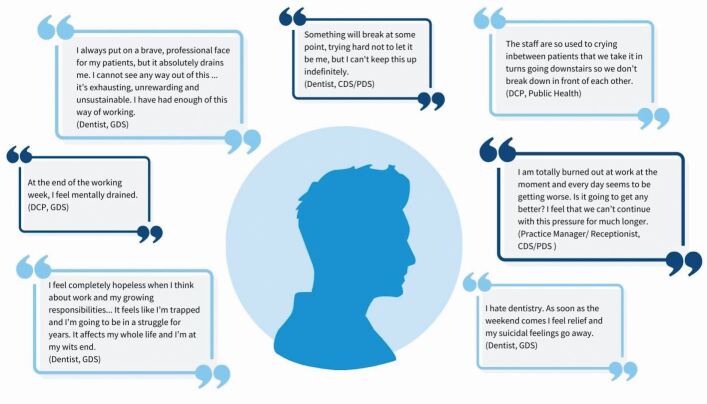
Quotes from respondents revealing depths of despair

The aim of this study was to identify and explore the factors that contribute to stress and burnout within the dental team. However, before presenting the results of the thematic analysis, we feel it is important to actively acknowledge and forefront the depth of despair which exists within some members of the dental profession. The extracts shown in Figure 1 illustrate the level of mental distress and despondency which some dental team members are currently experiencing.

**Fig. 2:**
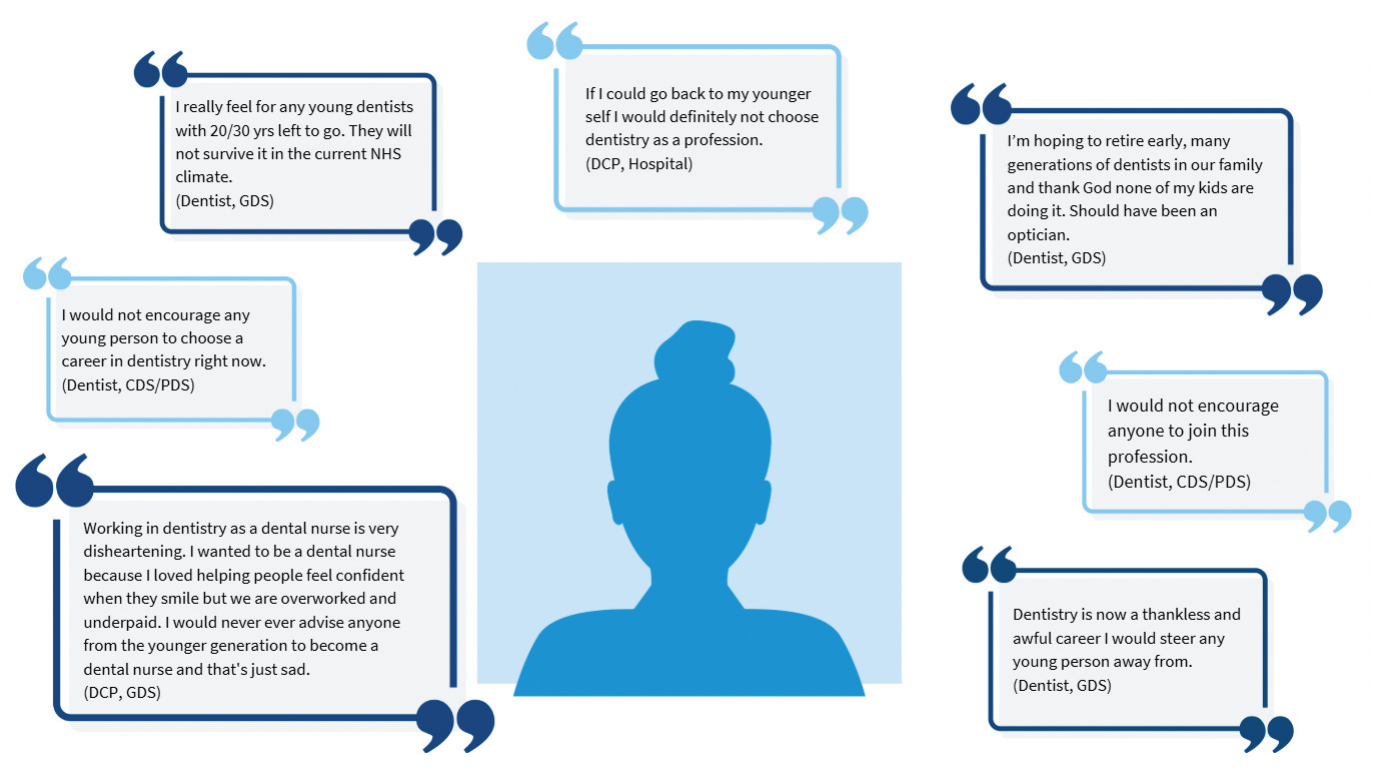
Quotes from respondents about recommending a career in dentistry

Several respondents expressed a negative view of dentistry as a career, with some actively discouraging young people not to join the profession (Fig. 2).

### Key stressors

Six themes were identified from the data: workload; NHS system; regulatory compliance, patient complaints and litigation; financial pressures; leadership and management; and self-worth.

#### Workload

Respondents frequently referred to an intolerable workload, which was described as steadily increasing with little sign of respite:

- ‘Patient numbers and demand are much higher than dentists are now able to cope with and it's affecting all levels of staff, from reception to surgery' (DCP, GDS independent).

Demand for services continued to rise, which led to long waiting times and additional pressure and stress on staff:

- ‘The demands on service are overwhelming. Pre-COVID, I worked 2-3 weeks ahead…I am now booked three months ahead. This has led to an incredibly stressful work environment' (dentist, GDS independent).

Issues around recruitment and retention, particularly in the NHS, placed additional pressure on remaining staff, causing further frustration and stress on all members of the dental team:

- ‘We are massively short-staffed (50% of normal staffing currently at my own clinic) but the volume of work remains the same, so the pressure on staff increases' (dentist, CDS/PDS).

#### NHS system

Working within the NHS was considered particularly stressful. The increasing demands on services, coupled to reduced capacity, was seen as a major contributing factor in stress and burnout:

- ‘The greatest stress frustration comes from high volumes of patients seen at an NHS practice and providing work to a quality I would like while working towards NHS UDA [units of dental activity] targets' (dentist, GDS independent).

Criticism of the NHS was universal, despite different contractual systems being adopted across each of the four UK countries. In England, the UDA was widely criticised, with frustration at the delays in implementing contract reform, which undermined any hope that change was imminent:

- ‘Feel under constant pressure and stress from NHS unachievable targets…[the] Government [is] not bothered or committed to funding so [it] seems [there is] no positive future for NHS dentistry' (dentist, GDS independent).

Disaffection with the NHS was a key driver towards a shift to the private sector, which was considered to offer a better working environment, less stress and greater autonomy:

- ‘I now work privately with children's NHS only. There is less pressure, less complaints and I am less stressed' (dentist, GDS corporate).

#### Regulatory compliance, patient complaints and litigation

Increasing levels of regulation, with a constant fear of complaints, litigation, or referral to the General Dental Council (GDC), were a recurring theme in relation to stress. There was widespread resentment at the time and effort required to demonstrate regulatory compliance, much of which was considered unnecessary:

- ‘The increasing nonsense regulations that don't improve patient care one bit that we all pretend to follow and the increasing complaint culture has sucked the fun out of dentistry' (dentist, GDS independent).

Regulatory bodies, such as the GDC or Care Quality Commission, were frequently referenced as being overzealous and unsympathetic, and viewed as responsible for creating a climate of fear which caused considerable stress:

- ‘The atmosphere from regulatory bodies, constant fear of disciplinaries, constant litigation fear and the feeling of being held to ransom by patients with axes to grind' (dentist, GDS corporate).

Patient complaints were considered one of the greatest stressors and appeared to be a constant worry for many:

- ‘I am constantly worried about receiving complaints' (dentist, GDS independent).

Respondents reported a significant increase in the number of complaints and felt this had escalated since the COVID-19 pandemic:

- ‘We have verbal complaints daily about appt [appointment] waiting times and written complaints have increased 200%! Prior to COVID we could go years without a written complaint' (practice manager/receptionist, GDS independent)

Part of the reason for a rise in complaints was reported to be a shift in patient expectations, which were often deemed to be unrealistic, particularly within the NHS:

- ‘I have always enjoyed my job but I feel patients are more angry, frustrated and worried than before and we seem to get much more moaning and complaints directed at us. We have worked tirelessly to try and catch up (while being short of staff and a dentist) but can't meet all patient expectations within an NHS system that is not fit for purpose now' (dentist, GDS corporate).

Strong views were expressed that delivering care to patients with high expectations, under NHS contractual arrangements with the overriding threat of regulatory non-compliance, created a difficult and stressful environment:

- ‘It's not patients or patients' expectations that keep us up at night. It's the fear of the reprisals that could come as a result of us not meeting what are essentially unrealistic expectations placed on us by the state and the patients' (dentist, GDS independent).

Complaints were a major stressor for all members of the dental team, but it was acknowledged that practice managers and receptionists were often the team members who had to deal directly with disgruntled patients:

- ‘It is practice managers and reception staff that are taking the brunt from patients' dissatisfaction, frustration and fear' (practice manager/receptionist, GDS corporate).

For dentists, the underlying worry from a complaint was the risk that it may escalate and lead to litigation or referral to the GDC, and this was referenced by multiple respondents:

- ‘Currently, threats of litigation and GDC referral terrify and isolate most GDPs [general dental practitioners] just trying to do their jobs' (dentist, GDS corporate).

Although an issue for all dental care professionals, litigation and referral to the GDC appeared to cause the greatest concern among dentists working in general dental services.

#### Financial pressures

Although dentistry can be professionally and financially rewarding for dentists, financial pressure, particularly when linked to NHS performance targets, was highlighted as a major cause of stress:

- ‘The stress of meeting targets and UDAs led me to leave a self-employed mixed practice for a salaried post where I don't have to worry about how much money I'm going to bring home each month. I also don't have to feel like I have to rush patients and cut corners' (dentist, CDS/PDS).

While some had left the NHS (GDS) to work in the salaried service, others had left to work in the private sector. Others expressed a desire to leave dentistry altogether but felt trapped due to their own financial obligations:

- ‘I'd leave if I could. Financially, not a possibility' (dentist, GDS independent).

Financial pressures, poor pay and feelings of being undervalued were frequently expressed by DCPs who were frustrated and resentful at how hard they had to work, while being poorly remunerated compared to many other occupations:

- ‘Wages poor and do not reflect amount of hard work and commitment of staff' (DCP, GDS independent).

#### Leadership and management

Many of the respondents cited frustration towards management at a practice or organisational level, which they felt contributed to the stress of the job:

- ‘I love my job and I am good at it. However, management is awful. No interest in staff. My job doesn't stress me. Management do!' (DCP, schools and nurseries).

This stress appeared to be related to a variety of issues, including lack of support and guidance, or due to perceived unfairness or inequality within the team:

- ‘There is huge lack of support and guidance in my post. The right things are said but never put into practice and I can say this with certainty as I have worked in post for many years' (DCP, CDS/PDS).

Although corporates were mentioned in relation to poor management, respondents were equally critical about principals within independent dental practices who failed to appreciate staff or associates:

- ‘I was a mixed NHS/private, high-grossing associate, people pleaser, did everything in practice, but felt unappreciated by principal' (dentist, CDS/PDS).

This was often closely aligned to poor organisational leadership, irrespective of the setting. If the team was not well led and functioning properly, members of the team were increasingly stressed and unhappy in their work:

- ‘I feel there is a total lack of effective leadership in the workplace…I'm well aware we all have a role to play in the management of our service delivery but there has to be a hierarchy of leadership overseeing services, with tasks delegated appropriately according to scope of practice and experience' (DCP, hospital).

#### Self-worth

A consistent theme throughout the data was the importance of feeling valued and appreciated at work. Some of this was related to external factors, such as remuneration, respect, gratitude, teamwork, good management and professional fulfilment. When respondents felt undervalued or underappreciated in their role, they felt unhappy, stressed and unfulfilled:

- ‘The morale of the dental team is in the toilet. We are underfunded and underappreciated and frankly we're sick of it' (dentist, GDS corporate).

For dentists, the frustration was often attributed to the system, in particular, the NHS:

- ‘I resigned in February…I felt stressed…I felt overworked and undervalued working for the NHS and so I am moving to private practice' (dentist, GDS independent).

For DCPs, the frustration was more often centred on the organisation, where they felt taken for granted and underappreciated:

- ‘Without the dental nurses, the whole practice could not function and I believe the dental nurses need the most support for their mental health, as they are the least paid but do most of the essential day-to-day jobs needed to run the practice' (DCP, GDS corporate).

Some struggled with a constant feeling of failure as they felt they had been unable to deliver the care their patients needed:

- ‘I go home knowing that I have failed my patients' (dentist, CDS/PDS).

Others felt trapped, or unable to escape:

- ‘I just want to leave and stop but I can't disappoint my parents or business partner' (dentist, GDS independent).

While others grappled with self-doubt:

- ‘Every day working in the dental industry is draining, burning me out and making me question my ability to do my job' (DCP, GDS corporate).

The importance of support was identified by several, and this was underlined by the various comments about loneliness and isolation:

- ‘Dentistry is a horrible, lonely profession' (dentist, GDS independent).

As previously highlighted, management had an important role in promoting teamwork and ensuring that individuals felt valued as members of the team. When this was not the case, individuals felt isolated and worthless:

- ‘I feel like I am alone, with no support…there is no motivation for a GDP working for the NHS to give more than necessary, as there is very low, if any, recognition…from the practices' (dentist, GDS corporate).

## Discussion

The qualitative findings from the MINDSET U.K. Survey 2023 provide further evidence that a significant proportion of the dental workforce are unhappy and unfulfilled in their role. What is most concerning is the level of despair among some respondents who appear to be suffering serious mental health problems, including anxiety, stress, burnout and even suicidal ideation. The dental literature has previously focused on the mental health and wellbeing of dentists rather than the wider dental team, a point highlighted within the previous GDC *Mental Health and Wellbeing in Dentistry: A Rapid Evidence Assessment* in 2021.^4^ The MINDSET U.K. Survey is the first study that we are aware of that has included the wider dental team and acknowledges the impact which stress and burnout can have on each and every member of the dental workforce. Our data indicate that all groups within dentistry are impacted adversely by stress and burnout, and although there are many similarities, some differences were identified which clearly necessitate further research.

Analysis of the quantitative data collected in the MINDSET U.K. Survey 2023 found that a large proportion of respondents across all dental groups reported heightened levels of stress, burnout and depression, with 61% experiencing high levels of emotional exhaustion.^19^ This is not unique to UK dentistry, and recent studies in Australia,^20^ Spain^21^ and the United States^22^ have also reported high levels of burnout, albeit within dental practitioners.

The qualitative data are particularly important as they offer additional insight into the stressors which are contributing to poor mental health in dentistry. An understanding of the stressors involved provides an opportunity to implement a range of measures to reduce or mitigate against their effects. This can be focused at a personal, organisational, or systems level,^23^ described by Gallagher as micro (relationships and personal factors), meso (related to workplace and job specification) and macro (the dental system, regulation, profession and society).^24^ Newton refers to this as the ‘me, we, they' approach.^25^ This simple description provides a helpful tool for reflecting on the role we each play in reducing stress through self-care, teamwork and influencing change at a systems level.

The contributing factors for dentists are reasonably well-documented in the literature^2^^,^^3^^,^^12^^,^^13^^,^^14^^,^^15^^,^^26^ and the results of the MINDSET U.K. Survey align closely with research published by Toon *et al.,* which in turn heavily influenced the GDC *Rapid Evidence Assessment.*^4^ Toon *et al.* reported that the highest levels of stress and burnout within UK dentists were among general dental practitioners working within the NHS,^27^ with the main contributory factors identified as ‘productivity stress, work content stress, patient-led stress and regulatory stress'.^27^

The results of our dental team survey indicate similar findings across all groups, highlighting the detrimental impact of increasing workload, exacerbated by difficulties around workforce recruitment and retention. The challenges of working within the NHS, with unrelenting demands on services, target-driven care and poor remuneration, created pressure and stress on all members of the dental team.

Stressors were reported across all workplace types but it is notable that several respondents reported relief on leaving the GDS and moving to the salaried service, or more commonly, the private sector. This resonated with the findings from Toon *et al.*, who reported that dentists working in the GDS were under particular stress.^27^ Private practice was considered a less stressful work environment, with a greater focus on person-centred care, less emphasis on performance targets, greater autonomy and better remuneration.

Remuneration was an area where differences were noted between the respective respondent groups. Dentists frequently focused on the lack of funding within the NHS and the impact which this had on the financial viability of the business. Rising costs and stagnant NHS funding causing increasing pressure on the profitability of practices led to significant stress and worry for practice owners. This aligned with the business-led stressors reported in the GDC *Rapid Evidence Assessment*.^4^ Although non-practice owners were aware of the funding issues, they were more concerned with the consequences - more work for less money. Depressed wages were frequently highlighted by members of the dental team in GDS settings who felt they were overworked and underpaid. Low income was a stressor but it also contributed to the feeling of being undervalued and underappreciated.

Patient complaints, although highlighted in all groups, appeared to cause different types of anxiety and stress dependent on the individual's role. Receptionists and practice managers were often responsible for managing patient complaints, which were seen as challenging, time-consuming and emotionally draining. For dentists, the potential consequences of complaints, with the threat of litigation or referral to the GDC, were reported as causing significant levels of anxiety for many. This was a key factor identified in the GDC *Rapid Evidence Assessment* and previously highlighted in several other studies.^2^^,^^12^^,^^14^^,^^15^^,^^27^

At a time when NHS dentistry is under significant pressure, with ongoing issues around workforce recruitment and retention, it is imperative that we have a healthy, happy and motivated workforce. Attrition of the workforce through career changes, early retirement, or ill health places significant strain and pressure on the remaining workers. This in itself causes additional stress and emotional exhaustion as individuals are asked to undertake extra duties or carry out unfamiliar tasks in different settings.^21^ Criticism of NHS dentistry is well-documented^2^^,^^12^^,^^27^ and our data would support the view that working in the NHS is a major contributing factor in terms of stress and burnout. For some, a change of work was not perceived as a viable option, which led to feelings of resentment and entrapment, resulting in further stress and unhappiness. For those who felt ‘trapped in the system', there was a feeling of failure or lack of professional fulfilment or personal accomplishment, which is considered a contributory factor in burnout. Suicidal thoughts were mentioned by some respondents, which is deeply concerning. This aligns closely with O'Connor's integrated motivational-volitional model where ‘defeat, humiliation and entrapment are the fuel upon which suicidal ideation thrives'.^28^

Considerable focus has been placed on increasing resilience within the profession^6^^,^^29^^,^^30^ to cope with the stress and strain of working in dentistry. A variety of organisations have stepped forward to offer advice and support for those struggling with their mental health. Organisations such as the NHS, British Dental Association, Dental Professional Alliance, GDC, indemnity organisations, Confidental and the Dentists Health Support Trust do fantastic work and offer a range of resources to help individuals manage the consequences of a career in dentistry.

The primary role of an oral healthcare professional is to focus on prevention of oral disease. Ironically, the approach towards mental health and wellbeing within dentistry is often reactive, with a focus on management of the consequences rather than addressing the underlying factors. The Mental Health Wellness in Dentistry Framework^31^ was developed to promote ‘a whole team approach' with a focus on ‘early intervention and safe signposting'.^31^ This initiative has raised awareness of mental health and wellness within the dental profession and encouraged dental teams to take a more proactive approach at both an individual and organisational level. It has been suggested that individual-level interventions may have limited value on a wide-scale within industry, without organisational change.^32^ Within dentistry, the influence of the system at a macro-level would seem to have a major impact, and without significant and urgent changes, the dental profession will continue to struggle.

### Limitations of this study

There are limitations to the study insofar as a non-probability convenience sample should not be considered to be representative. The method of collecting qualitative data in this study via a free-text response may be viewed as lacking depth compared to other qualitative methods. However, it is also possible that the anonymous nature of the survey allowed respondents to be more honest about their emotional states than might otherwise have been the case. It is also important to acknowledge that although many of the stressors identified within this study will affect all members of the dental team, there are likely to be nuanced differences between groups. The data captured on specific dental team roles had insufficient granularity and it is clear that more research needs to be undertaken to explore this topic in more detail to include all members of the dental team.

## Conclusion

The qualitative findings from the MINDSET U.K. Survey 2023 provide further evidence of the strain which many members of the dental team are under and offer insight into the key stressors implicated. Anxiety, stress and burnout have a deleterious impact on the ability of the dental profession to deliver high-quality oral healthcare. If the underlying issues are ignored, high levels of stress and burnout in dentistry will continue, particularly within the NHS, with serious consequences.

There has been an increasing focus on mental health in dentistry over recent years but much of the work is based on a reactive approach, offering support and advice on ways to manage stress or increase resilience. Advice and support have also tended to focus on the dentist, rather than the wider dental team. A preventive approach needs to be adopted but simply focusing on the resilience of the individual or the team is misguided and unsustainable. The main stressors identified within our study can be largely attributed to macro-level factors. This suggests that the major stressors to UK dental teams sit predominantly outside of their locus of control and require prioritisation of policy and system-wide interventions over individual and team-based solutions. It is critical that dental regulators, policymakers and NHS commissioners acknowledge their role in contributing to stress within the profession and actively seek to change this.

## Resources

The British Dental Association has compiled a selection of resources to help support wellbeing, build resilience and connect with colleagues: https://www.bda.org/advice/wellbeing/.

## Data Availability

The dataset presented in this article is controlled by NHS Education for Scotland. Access to the data is subject to approval and a data sharing agreement. Access requests should be directed to sdpbrn@nes.scot.nhs.uk.
